# Reconsideration of Laparoscopic Cholecystectomy

**DOI:** 10.5402/2011/827465

**Published:** 2011-06-30

**Authors:** Kazuhiko Kasuya, Takao Itoi, Takaaki Matsudo, Bunsoh Kyo, Yasushi Endo, Takahisa Ikeda, Yuichi Nagakawa, Yoshiaki Suzuki, Motohide Shimazu, Tatsuya Aoki, Akihiko Tsuchida

**Affiliations:** ^1^Department of Surgery, Tokyo Medical University, 6-7-1 Nishishinjuku Shinjuku-ku, Tokyo 160-0023, Japan; ^2^Department of Internal Medicine, Tokyo Medical University, Tokyo 160-0023, Japan; ^3^Department of Surgery, Hachioji Medical Center, Tokyo Medical University, Tokyo 160-0023, Japan

## Abstract

We describe the surgical method of cases showing a distended gallbladder. Because the most important thing does not cause biliary tract injury, it is to find orientation carefully. The frequency of incidental gallbladder cancer was in 7 (0.7%) of the 983. Only cholecystectomy is necessary to be performed for Tis or T1 cancer, and surgery has to be changed to radical surgery for T2 cancer or deeper invasion. Laparoscopic cholecystectomy is already an established standard operation. In the presence of acute or severe chronic inflammation, special attention should be paid to these points.

## 1. Introduction

Laparoscopic surgery has rapidly spread and has been applied to surgery of not only the gastrointestinal tract, but also the liver, gallbladder, and pancreas. Laparoscopic surgery was initially applied to cholecystectomy, and laparoscopic cholecystectomy is a standard operation at present. This surgery can be readily performed in typical cases showing mild inflammation. However, in the presence of acute inflammation as a complication, there are problems such as tissue congestion, its susceptibility to bleeding, and distension of the gallbladder. When there is severe chronic inflammation after repeated inflammation, fibrosis from the gallbladder neck to Calot's triangle is marked, and orientation is difficult in some cases, increasing the surgical difficulty. 

We describe laparoscopic cholecystectomy with a high difficulty level, complications, and gallbladder cancer as a complication that could not be preoperatively diagnosed.

## 2. Laparoscopic Cholecystectomy for the Gallbladder with Acute or Chronic Inflammation

### 2.1. Cases Showing a Distended Gallbladder

In patients with acute cholecystitis or stone incarceration in the gallbladder neck, the gallbladder is first punctured and the contents are aspirated. When the gallbladder is filled with stones, the gallbladder is incised, and as many stones as possible are removed from it.

### 2.2. Cases Showing Marked Fibrosis in Calot's Triangle

After the neck is dissected as extensively as possible, dissection is started from the gallbladder bed at the gallbladder fundus. The body and fundus of the gallbladder are dissected to a certain extent, and the gallbladder is laterally pulled, and when the neck is observed again, orientation is often easy.

### 2.3. Cases with Confluence Stones

When Endoscopic nasobiliary drainage (ENBD) or endoscopic nasogallbladder drainage (ENGBD) catheter has been placed before the operation, a clip for marking is applied to the site that appears to be the cystic duct, and cholangiography is performed early to identify the cystic duct site.  After the stone location has been clarified, a longitudinal incision is made on the gallbladder side of the stone, and the stone is removed. When the cystic duct is thickened, cutting using an automatic cutting device is safe and straightforward.

### 2.4. Cases Requiring the Dissection of Severe Adhesion

For the dissection of severe adhesion of the greater omentum and resection of the thickened gallbladder wall, the use of ultrasonic cutting and coagulation systems such as ultrasonic laparoscopic coagulating shears (LCS), SonoSurg (Olympus, Tokyo, Japan), and Harmonic Scalpel (Johnson & Johnson, Cincinnati, OH, USA) can reduce bleeding and are useful. The Cavitron Ultrasonic Surgical Aspirator (CUSA; Valley lab Inc., Boulder, CO, USA) is useful for the dissection of the cystic artery and cystic duct in some patients.

When dissection is difficult even using these methods, resection by open surgery is indicated.

## 3. Major Complications

### 3.1. Biliary Tract Injury

The treatment of bile duct injury differs according to the degree of injury and timing of its diagnosis. In cases with a bile duct incision, suturing and closure of the incised area and bile drainage are performed. In cases with complete bile duct transection, cholangiojejunostomy is often performed. When such transection is noticed during the operation, treatment is relatively straightforward, and the prognosis is also favorable. However, when it is noticed after the operation, reoperation is difficult, and biliary stricture or choledocholithiasis sometimes develops. Biliary stricture is the most serious complication of laparoscopic cholecystectomy.

### 3.2. Bleeding from the Gallbladder Bed

Deep cutting of the gallbladder bed damages the liver parenchyma, inducing bleeding. In particular, when peripheral branches of the middle hepatic vein are present in contact with the gallbladder bed, massive bleeding sometimes occurs. Pressure application with the use of absorbable local hemostatic agents is useful.

## 4. Incidental Gallbladder Cancer

### 4.1. Usefulness of Intraoperative Rapid Pathological Diagnosis

Port site recurrence or abdominal dissemination after laparoscopic surgery for gallbladder cancer not preoperatively diagnosed has been reported [1]. Advanced gallbladder cancer sometimes recurs even after re-operation. We perform intraoperative rapid pathological diagnosis using frozen sections in all patients undergoing laparoscopic cholecystectomy. Of 990 consecutive patients who underwent rapid pathological diagnosis, 983 were diagnosed with no tumorous lesions as complications, and 7 were diagnosed with cancer. Postoperative permanent specimens revealed cancer in 4 (0.4%) of the 983 patients who were diagnosed with benign lesions, and all 7 patients who were diagnosed with cancer by intraoperative rapid pathological diagnosis. Only cholecystectomy was performed for Tis or T1 cancer, and surgery was changed to radical surgery for T2 cancer or deeper invasion [2, 3].

## 5. Present Status

Present status of laparoscopic cholecystectomy was reported by the Japan Society for Endoscopic Surgery [4].

- Endoscopic surgery in the abdominal surgery field was initiated in about 1990, and the number of cases treated with this technique has steadily increased, reaching more than 60,000 in 2009.
- In gallbladder diseases, the annual number of cases treated with endoscopic surgery was about 26,000 in 2009, accounting for about 43% of abdominal surgeries.
- The method of inserting the first trocar is the small incision method in most cases.
- Laparoscopic cholecystectomy was performed in 142 patients with gallbladder cancer, which accounted for about 0.5%.
- The laparoscopic versus open cholecystectomy ratio is 8 : 2.
- Bleeding during surgery occurs in about 0.4%, biliary tract injury in about 0.6%, and injury to other organs in 0.2%.
- Conversion to open surgery is observed in about 6%.

## 6. Conclusion

Laparoscopic cholecystectomy is already an established standard operation. However, once biliary stricture due to intraoperative biliary tract injury occurs, treatment, irrespective of whether it is conservative or surgical, requires a few months–years, markedly impairing the patient's quality of life. In addition, an accurate preoperative diagnosis of the presence/absence of gallbladder cancer in all cases is impossible even using the latest diagnostic imaging techniques, and the possibility of gallbladder cancer as a complication should always be taken into consideration. The incidences of both biliary stricture and gallbladder cancer are high in laparoscopic cholecystectomy in the presence of acute or severe chronic inflammation, and special attention should be paid to these.
